# Low Temperature Plasma Jet Treatment Promotes Skin Wound Healing by Enhancing Cell Proliferation via the PI3K‐AKT and AMPK Pathways

**DOI:** 10.1111/iwj.70213

**Published:** 2025-02-11

**Authors:** Yuehan Zhu, Xinrong Lian, Kaici Li, Jingya Zhang, Wenjing Wu, Xinhua Zhang, Jin Zhang

**Affiliations:** ^1^ College of Biological and Chemical Engineering Jiaxing University Jiaxing China; ^2^ College of Life Sciences and Medicine Zhejiang Sci‐Tech University Zhejiang China; ^3^ School of Photoelectric Engineering Changzhou Institute of Technology Changzhou China; ^4^ Jiaxing i‐Bio Biotechnology Co. Ltd Jiaxing China

**Keywords:** AMPK, low temperature plasma jet, PI3K‐AKT, skin wounds, treatment

## Abstract

Low temperature plasma jet (LTPJ) treatment can promote skin wound healing, but the underlying molecular mechanisms remain poorly understood. In the present study, we verified the effect of LTPJ in accelerating wound healing and investigated its underlying mechanism. With mouse model, two full‐thickness dermal wounds were created in each mouse (*n* = 8). One wound underwent LTPJ treatment for 10 min, while the other wound without LTPJ treatment served as a control. The percentage of wound closure and collagen content in epidermis increased significantly, which indicated that LTPJ treatment significantly enhanced wound healing through contraction. RNA‐seq analysis was conducted to understand the underlying mechanisms. A total of 77 differentially expressed genes (DGEs) were identified. GO and KEGG pathway enrichment analyses revealed that the DGEs were mainly related to the collagen‐containing extracellular matrix, cell cycle, PI3K‐AKT signalling pathway and AMPK signalling pathway, which are known to be related to wound healing. HaCaT keratinocytes were used to study LTPJ effects on cell proliferation in vitro. In agreement with the in vivo results, the in vitro datas also demonstrated that LTPJ treatment affected the activity of the PI3K‐AKT and AMPK pathways. Our findings suggest that LTPJ treatment promotes skin wound healing by inducing genes associated with wound healing, promoting PI3K‐AKT signalling, and suppressing the AMPK signalling pathway.

**Summary**

- Low temperature plasma jet (LTPJ) generated by a hand‐held device accelerated skin wound healing in mice model.
- LTPJ treatment promoted collagen formation in skin wounds.
- LTPJ treatment increased the activity of PI3K‐AKT pathway and decreased the activity of AMPK pathway both in vivo and in vitro.

## 1 Introduction

As the body's largest organ, the skin serves various vital functions such as providing a physical barrier against pathogens, as well as performing absorption, secretion and excretion, thermoregulation, sensory, immune, respiratory and endocrine functions [1]. Due to its exposed location, skin is susceptible to various factors that can result in tissue damage and the onset of trauma [2]. Wound healing comprises a cascade of multiple biological and biochemical processes, which consist of a series of phases involving blood clotting, inflammation, cell proliferation, migration and differentiation, leading to the formation of granulation tissue, wound contraction, followed by final tissue restoration and re‐modelling [3]. Debridement, antiseptics, wound dressings and negative pressure therapy are common wound treatment methods, especially for chronic or non‐healing wounds [4, 5]. However, painful treatment, repeated medical visits and the great expenses involved in treatment lead to poor patient compliance [6, 7]. In addition, the emergence of antibiotic resistance further increases delays in the healing process. Thus, researchers are increasingly exploring new wound treatment methods and optimising treatment schemes to improve the efficacy of wound treatment.

Physically, plasma is metastable form of matter consisting of electrons, ions, photons and electromagnetic fields [8]. Plasma can be divided into thermal plasma (equilibrium plasma) and low‐temperature plasma jets (LTPJ; non‐equilibrium plasma) [9, 10]. Thermal plasmas are not suitable for use on living cells, tissues or temperature‐sensitive medical devices because they can reach very high temperatures [11]. LTPJ operates at temperatures similar to those of the human body (< 40°C) and produces several active substances such as electrons, ions, free radicals, UV light, heat, electric fields, etc. [12] One of the unique features of LTPJ is the possibility for local on target treatment, whether by delivering plasm effluent or direct plasma treatment [13, 14, 15]. Based on its painless nature, LTPJ is more suitable for dealing with cells and animal tissues. LTPJ technology has rapidly advanced and has found numerous applications in medicine, including skin decontamination and wound healing [16, 17]. Multiple research groups have confirmed the positive effects of LTPJ on skin wound healing and skin aesthetics [18, 19, 20, 21]. Additionally, LTPJ treatment is potentially a safe technique to decrease bacterial load of chronic wounds in numerous clinical trials [22, 23]. However, these devices are still in an early stage of development, and the mechanisms underlying their beneficial effects remain poorly understood.

In this study, our group applied a hand‐held LTPJ device, which utilises air as the working gas, to treat skin lesions in a mouse wound model, resulting in the promotion of wound healing. Subsequently, we performed a comprehensive transcriptome analysis of healing skin tissue with and without LTPJ treatment, using RNA sequencing technology (RNA‐Seq). Gene ontology analysis revealed that LTPJ treatment led to the differential expression of genes related to biological processes such as the collagen containing extracellular matrix, cyclin‐dependent protein kinase holoenzyme complex, cell division and cell viability. Moreover, the differentially expressed genes were mainly enriched in the PI3K‐AKT and AMPK pathways. Finally, similar results were obtained and verified using in vitro cell‐culture experiments, providing a new and effective method for the treatment of skin wounds.

## 2 Materials and Methods

### 2.1 A Hand‐Held Low‐Temperature Plasma Jet (LTPJ) Device

The hand‐held LTPJ device was developed by Enqi Zhang (Patent No. ZL 201921884898.8 and ZL 201920229222.9). Briefly, it utilises an intermediate frequency alternating high voltage electric field to ionise the air and generate a LTPJ. Compared with other plasma jet equipment, our device uses air as carrier gas instead of an inert gas, which makes it environmentally friendly and easy‐to‐use with no need for gas cylinders (Table S3). Furthermore, it is a hand‐held device with a weight of 600 g, which is very convenient for clinical application.

### 2.2 Wound Healing Model

All the animal experiments were approved by the Jiaxing University Experimental Animal Welfare Ethics Committee. Surgeries and sacrifice were performed under anaesthesia, and all efforts were made to minimise suffering. Sodium pentobarbital (Merck, Germany) was injected for preanesthesia. Then, respiratory anaesthesia was maintained using isoflurane liquid (EZVET, China). All methods used in this study were in accordance with ARRIVE guidelines for the reporting of animal experiments.

Eight six‐week‐old male C57BL/6 mice were used in the experiment. And the mice were purchased from Hangzhou Animal Experimental Center. All the animals were acclimated to the environment for a week before the experiment began after they were purchased. Animals were housed in a temperature‐controlled location with a 12 h light–dark cycle; they had free access to water and standard rodent chow. Animal protocols were approved in accordance with the guidelines established by the Research Animal Care Committee of Jiaxing University. Before wounding, anaesthetise mice with isoflurane (30 mg/kg), and then proceed to prepare the skin immediately. We used an electric hair clipper to remove the hair from their backs. On the same day as the skin preparation, two full‐thickness excisional wounds with a diameter of 6.0 mm were created on the dorsal back of each mouse using a punch biopsy tool (GSK) under sterile conditions. After constructing the skin wound model in the mice, we immediately performed plasma irradiation treatment on one of the wounds. One excision was exposed to LTPJ for 10 min at a distance of 2.0 cm as the treated group, and the other excision on the same mouse was left without treatment as the control group. In summary, the skin preparation, wound treatment, and plasma treatment are carried out on the same day. The wound area is measured and recorded as Day 0. Subsequently, the wound area is measured and recorded daily. When the percentage of wound closure reaches around 90%, euthanize the mice and take the skin from the original wound site for subsequent experiments (Figure S3).

### 2.3 Wound Healing Analysis

Digital images of the wounds were captured using a digital camera on days 0, 5 and 7 following the creation of the wounds. The dimensions of the wounds were measured by determining their horizontal and vertical radii. The percentage of wound closure was calculated by comparing the size of the wound at the specified timepoints to the initial size on the day of wounding.

### 2.4 Histological Staining and Analysis

The skin samples were preserved in a solution of 10% paraformaldehyde (in phosphate‐buffered saline at pH 7.4) and subsequently embedded in paraffin. A set of paraffin sections was stained with haematoxylin/eosin (H&E) and Masson's stain. Quantitative image analysis was conducted on the sections stained with Masson's stain to assess collagen deposition. Three sections were randomly selected from each of the 8 mice and scanned using a 20× objective on a DFC7000 T microscope (Leica, Germany). The recorded images were then used for further analysis [24, 25].

### 2.5 RNA‐Seq Analysis

RNA samples from each sample were extracted using TRIzol Reagent (Invitrogen, USA) in three independent biological replicates. BGI (Beijing Genomics Institute, China) conducted the sequencing procedure. The quality of the RNA was assessed using an Agilent 2100 instrument (Agilent Technologies Inc. USA), a NanoDrop 2000 (Thermo Fisher Scientific Inc. USA), and agarose gel electrophoresis. Three replicate RNA samples were used for constructing cDNA libraries. Specifically, oligo‐dT magnetic beads were employed to enrich mRNAs with poly A tails. The target RNA was fragmented and reverse‐transcribed into double‐stranded cDNA (ds‐cDNA) using N6 random primers. Subsequently, the ligation products were amplified using two specific primers. The PCR products underwent heat‐denaturation, and the single‐stranded DNA was circularised using a splint oligo and DNA ligase. Finally, transcriptome sequencing was performed on the cDNA library by employing a BGISEQ‐500 RNA‐Seq platform. The sequencing generated 50‐bp single‐end reads (SE50) [24].

### 2.6 Gene Expression Analysis

The FPKM (fragments per kilobase of transcript per million mapped reads) value and the DEGseq algorithm were utilised to assess the levels of gene expression and identify differentially expressed genes (DEGs), respectively. The criteria for filtering DEGs were fold‐change > 1 and FDR < 0.05. Hierarchical clustering analysis of DEGs was conducted using Cluster 3.0 with the average linkage and Euclidean distance metric. The results were visualised using Java TreeView (version 1.1.6r2, Stanford University).

### 2.7 Functional Enrichment Analysis

Gene ontology (GO) analysis and pathway analysis of the differentially expressed genes (DEGs) were performed using Gene Ontology (http://www.geneontology.org/) and KEGG (http://www.genome.jp/kegg/), respectively. Fisher's exact test was employed to identify the significant GO categories or pathways. Terms with corrected *p*‐values less than 0.05 were considered significant or enriched terms.

### 2.8 Cell Culture

Immortalised human HaCaT keratinocytes were cultured in Dulbecco's modified Eagle's medium (i‐Bio Biotechnology Co. Ltd. Jiaxing, China) supplemented with 10% fetal bovine serum (i‐Bio Biotechnology Co. Ltd. Jiaxing, China), 1% antibiotic–antimycotic solution (100×) at 37°C with 5% CO_2_. When HaCaT keratinocytes density reaches approximately 80%, LTPJ treatment is carried out. During LTPJ processing, the distance of cold plasma jet to media level was 2 cm and the irradiation time was 5, 30 or 60 s. After 24 h of LTPJ irradiation, collect the cells and perform subsequent detection. The experiment was repeated at least three times.

### 2.9 Proliferation Assay

Following 24 h after LTPJ exposure for 15, 30, or 60 s, HaCaT cell proliferation was determined using a CCK8 kit and EDU assay.

For EDU assay, 10 μM 5‐ethynyl‐2′‐deoxyuridine (EdU, RiboBio, Guangzhou, Guangdong, China) was added into the growth medium and incubated for 3 h. Fixation, permeabilization and EdU staining were done according to the manufacturer's protocol. Cell nuclei were counter‐stained with Hoechst 33342 at a concentration of 5 μg/mL for 10 min. Then, EdU‐positive cells were visualised under a fluorescence microscope (Nikon, Tokyo, Japan) to calculate the ratio of EdU‐positive cells (EdU‐stained cells/total cells).

Adipocytes were seeded into 96‐well plates at 5 × 10 [3] cells per well in 100 μL of growth medium. At 24 h after LTPJ exposure, the CCK‐8 kit (Beyotime, Shanghai, China) was used to detect cell proliferation according to the manufacturer's instructions.

### 2.10 10 Quantitative Real‐Time PCR

The reaction mixture consisted of nuclease‐free water (3.4 μL), forward primer (0.3 μL), reverse primer (0.3 μL), SYBR mix (5 μL) and 1 μL of the sample, making a total volume of 10 μL. The PCR temperature program included an initial denaturation step at 95°C for 2 min, followed by 40 cycles of 95°C for 2 s, 60°C for 20 s and 70°C for 10 s. The Ct value was calculated automatically using the default settings, with three technical replicates per sample (*n* = 3). GAPDH was used as the reference gene for real‐time PCR. The primer sequences are listed in Table S1 [25].

### 2.11 11 Western Blot Analysis

Total protein was extracted using RIPA buffer (Beyotime, Shanghai, China) supplemented with protease inhibitors (Pierce, Bradenton, Florida, USA) (*n* = 3). The lysates were centrifuged at 1360 g for 7 min, and the supernatant was then boiled in sodium dodecyl sulfate (SDS) loading buffer (Beyotime, Shanghai, China) for 10 min. After separating on 12% acrylamide SDS‐PAGE gels, the protein bands were transferred onto a polyvinylidene difluoride membrane (CST, Danvers, Massachusetts, USA). The membrane was blocked in 5% defatted milk and incubated with primary antibodie at 4°C for 12–14 h. The primary antibody was diluted in accordance with the corresponding ratio by using primary antibody dilution solution (Beyotime, Shanghai, China), followed by horseradish peroxidase‐conjugated secondary antibodies. After incubation with primary and secondary antibodies, the membrane was washed six times with TBST for 5 min each time. Specific bands were visualised using a chemiluminescence reagent (Millipore, Massachusetts, USA) and analysed using Quantity One 4.6.3 Image software, as previously described [25]. GAPDH was used as the reference gene and this gene was expressed stably during LTPJ treatment process (Figure S4). Antibody information is shown in Table S2.

### 2.12 1Statistical Analysis

All data were derived from at least three independent experiments and presented as means ± SD. Individual comparisons were assessed by multiple *t*‐tests. Differences with *p*‐values < 0.05 were considered statistically significant.

## 3 Results

### 3.1 LTPJ Accelerated Skin Wound Healing

**FIGURE 1:**
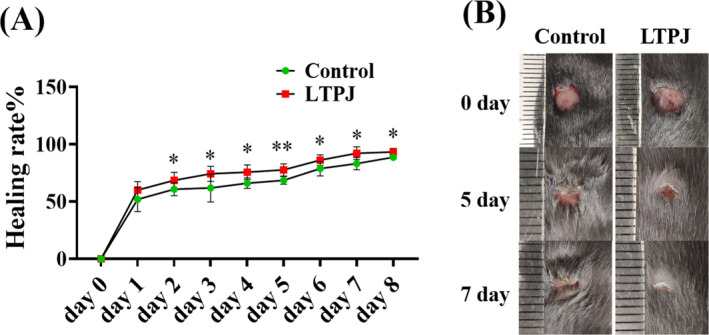
LTPJ promoted skin wound healing. (A) Quantification of the wound area in control and LTPJ treatment groups. (B) Gross observation of the wound healing process on days 0, 5 and 7 in the control and LTPJ treatment groups. Mean ± SD, *n* = 8, *p < 0.05, **p < 0.01.

The effects of LTPJ on wound closure were examined using full‐thickness dermal wounds in a murine model. In the study, a total of 8 mice were subjected to the wound creation process. The initial body weight of the mice in the experiment is 21.7 ± 0.62 g (Table S4). In studies of wound healing, wound contraction is indeed an important factor, as it helps to reduce the area of the wound, thus promoting the healing process. Therefore, when evaluating the effectiveness of wound healing, the degree of wound contraction is typically included in the statistical analysis. Based on the major and minor lengths of each wound, percentage of wound closure were calculated on each follow‐up day. Then, perform comparative statistical analysis between the LTPJ treatment group and the control group every day. LTPJ treatment showed beneficial effects on wound healing from day 2. The percentage of wound closure in the control group increased from 64% to 90% between days 2 and 8, while the treatment group increased from 68% to 93% (Figure 1A). The wound healing process was grossly examined on days 5 and 7 post‐wounding (Figure 1B). These data illustrate the wound healing effect of LTPJ treatment in the remodelling phase of wound healing.

### 3.2 LTPJ Promoted Collagen Formation

**FIGURE 2:**
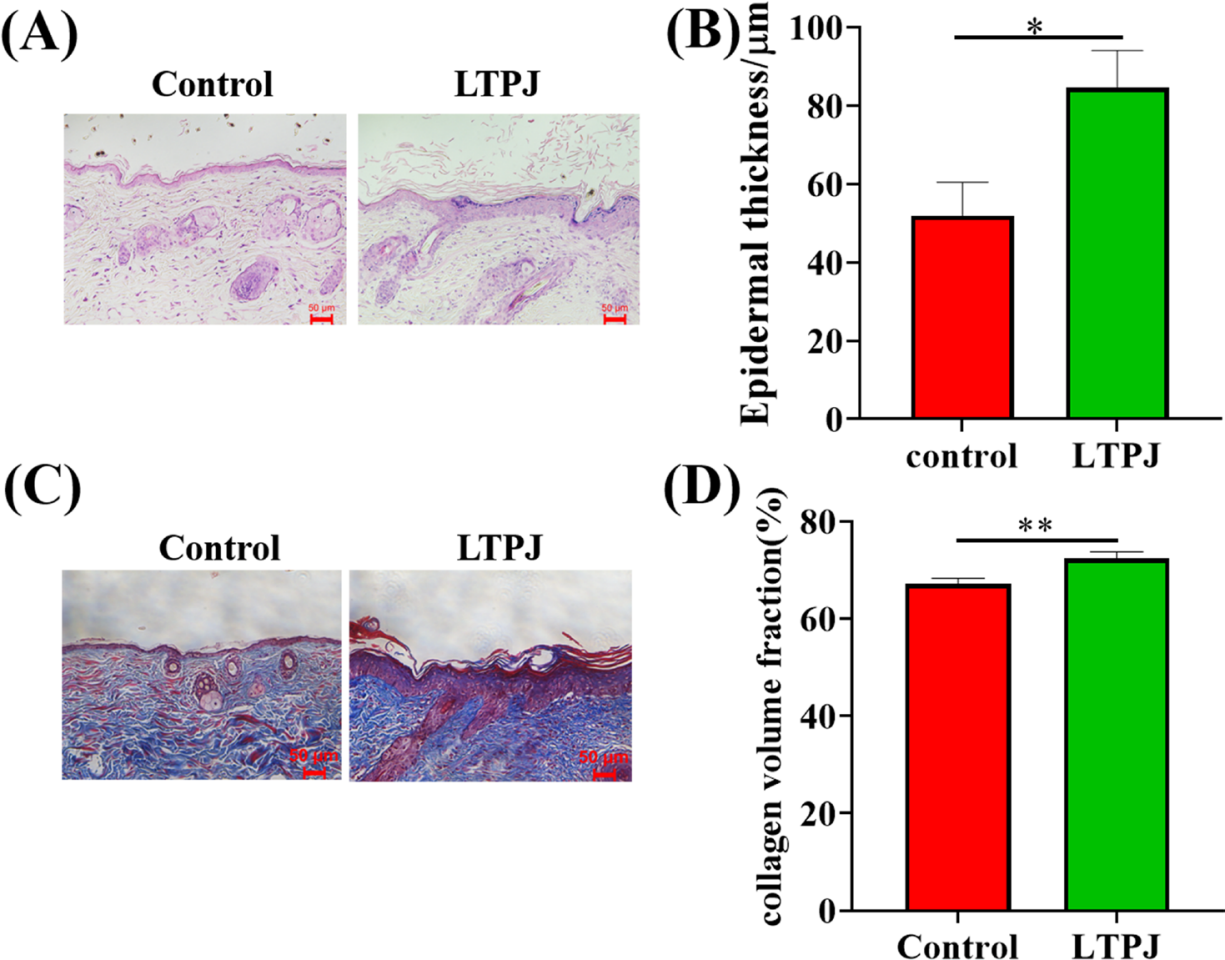
Representative Haematoxylin–Eosin and Masson's trichrome staining of wound tissue sections. (A) HE staining. (B) Quantitative analysis of epidermal thickness in control and LTPJ treated wounds on days 8 after the wounding. (C) Masson's trichrome staining. (D) Quantitative analysis of collagen staining (blue) density within the scar tissue. Mean ± SD, *n* = 8, *p < 0.05, **p < 0.01.

On the 8th day, pathological sections of the healing skin wounds were stained using H&E staining. Compared with the control group, the epidermis layer in the LTPJ‐treated skin samples was thicker. (Figure 2A and Figure S1).

The distribution of collagen within the wound area was assessed using quantitative image analysis of sections dyed with Masson's trichrome stain. Collagen formation was enhanced on day 8, while higher collagen deposition (*p* < 0.05) was found in the LTPJ‐treated group than in the control group (Figure 2B).

### 3.3 RNA‐Seq Identified the Differentially Expressed Genes in Healing Skin Tissue With and Without LTPJ Treatment

**FIGURE 3:**
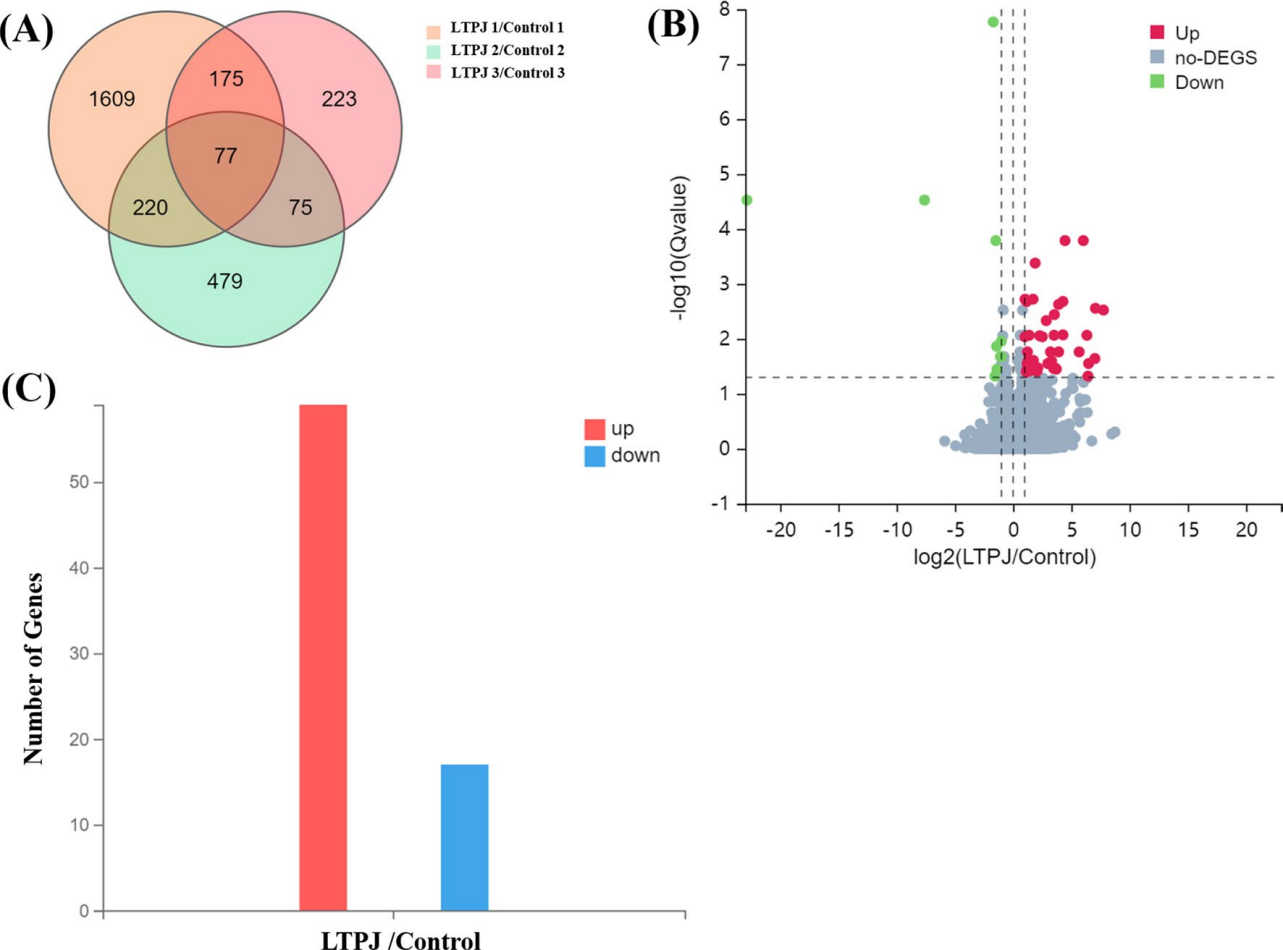
RNA‐Seq analysis of gene expression in the control and LTPJ treatment groups. (A) Venn diagram and (B) volcano plot showing the differentially expressed genes (DEGs) in wound tissue after LTPJ treatment. (C) Quantitative comparison of the up‐ and downregulated genes. *n* = 3.

There was a total of 77 differentially expressed genes (DEGs) between the LTPJ and control groups (Figure 3A), 58 of which were up‐ and 19 downregulated (Figure 3B,C).

**FIGURE 4:**
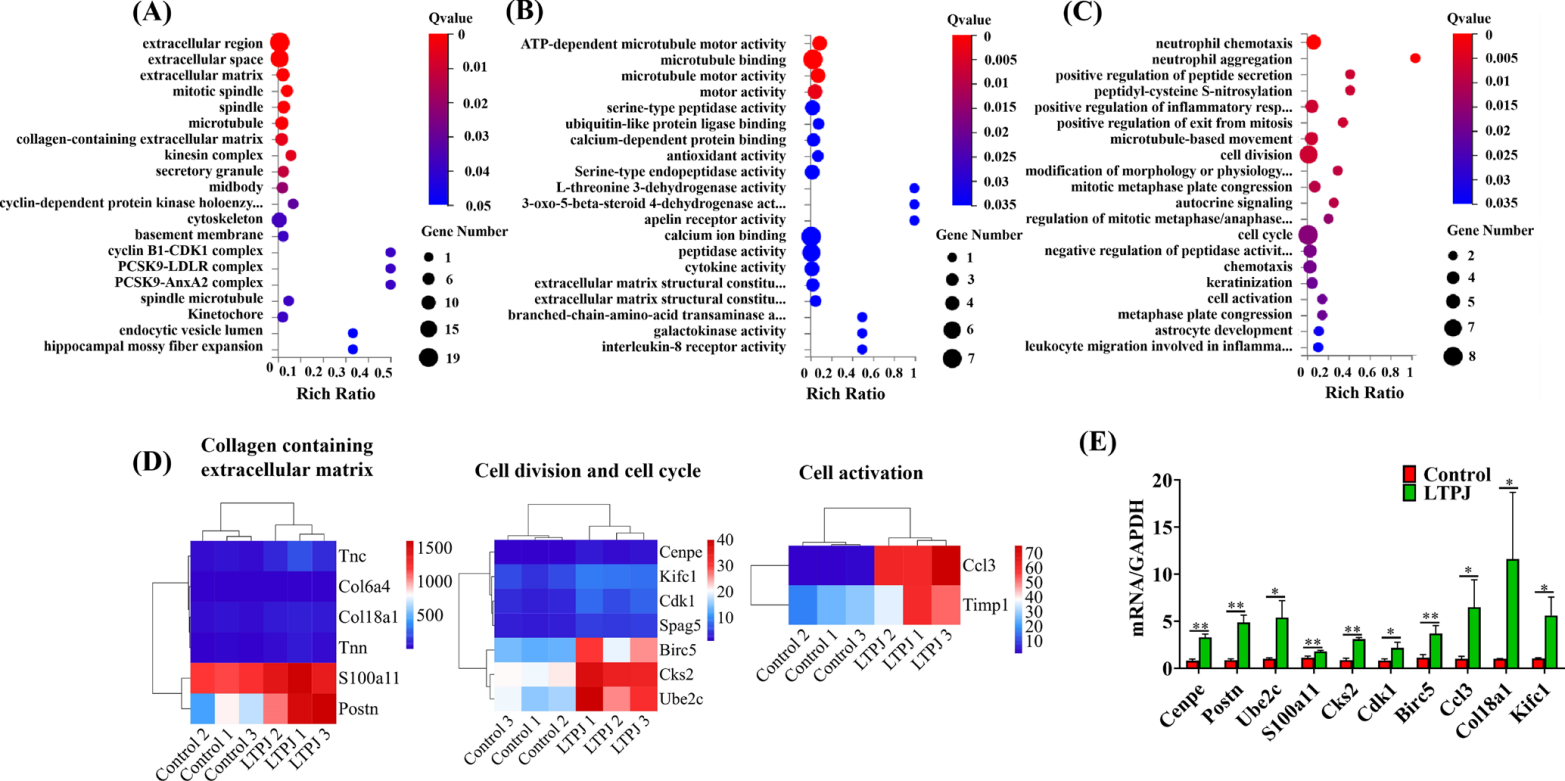
Gene ontology analysis of the differentially expressed genes. The results are summarised in the following three main categories: biological process (A), molecular function (B) and cellular component (C). Analysis of DEGs related to the collagen‐containing extracellular matrix, cyclin‐dependent protein kinase holoenzyme complex, cell division and cell viability using RNA‐Seq (D) and qPCR (E). Mean ± SD, *n* = 3, *p < 0.05, **p < 0.01.

GO analysis was preformed to further understand the biological functions of the DEGs in the skin healing process. The results showed that the upregulated DEGs were significantly enriched in biological functions related to the collagen containing extracellular matrix, cyclin‐dependent protein kinase holoenzyme complex, cell division and cell viability (Figure 4A–C). To validate the findings from RNA‐Seq, 11 genes related to the above biological functions were chosen for quantitative real‐time PCR (qRT‐PCR) analysis. The results obtained from RNA‐Seq and qRT‐PCR experiments are depicted in Figure 4. The expression patterns of the 11 tested genes were consistent between RNA‐Seq and qRT‐PCR (Figure 4D,E). These data confirmed that the results of RNA‐Seq analysis were indeed reliable indicators of overall changes in gene expression, indicating that the RNA‐Seq data were reliable and accurate.

### 3.4 Analysis of Pathways Related to the DEGs

**FIGURE 5:**
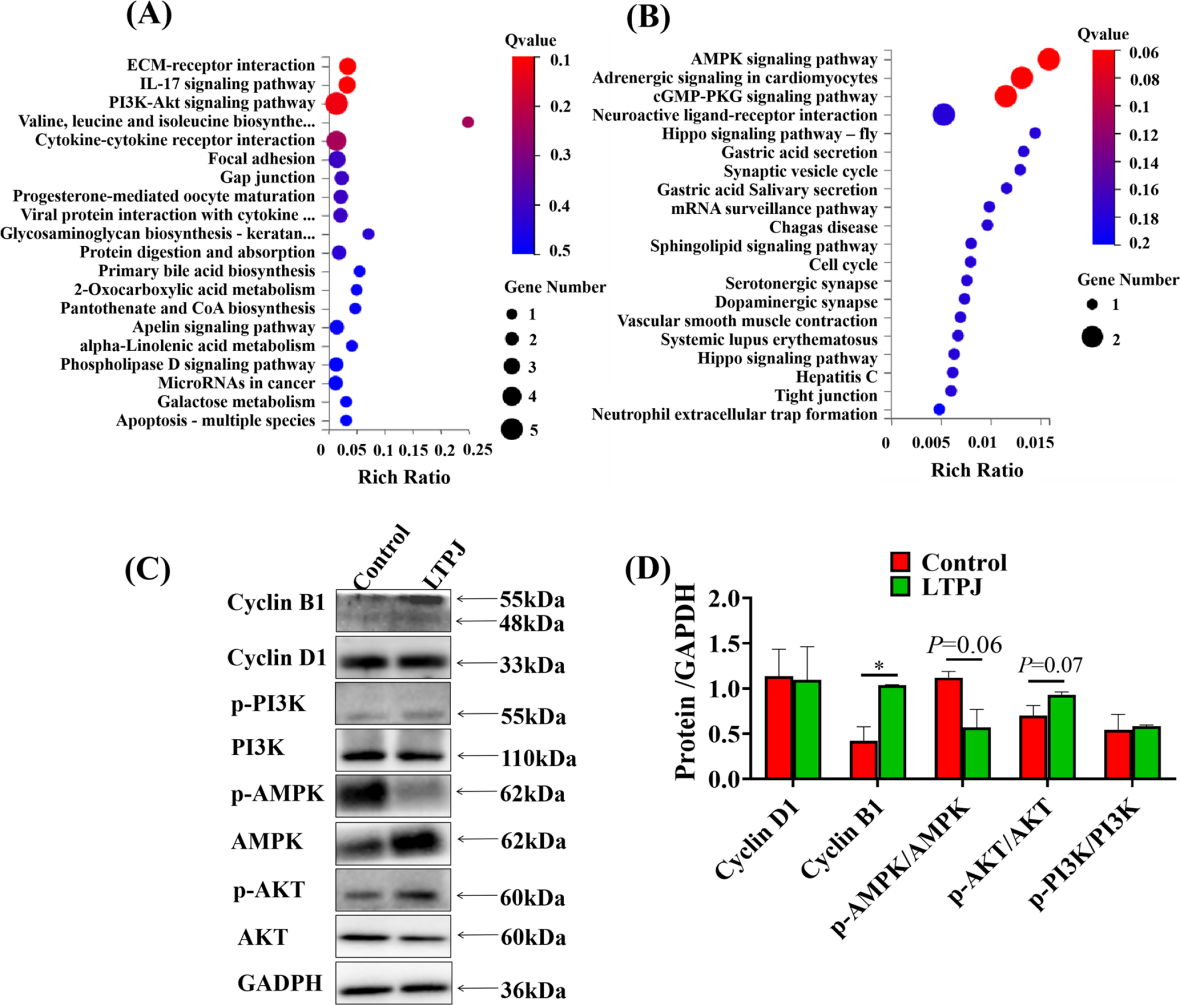
KEGG pathway analysis for differentially expressed genes. Enriched signalling pathways among the upregulated (A) and downregulated DEGs (B). (C) Western blot validation of signalling pathway activity. The grouping of blots cropped from different parts of the different gel. The data represent the means ± SD. *n* = 3, *p < 0.05, **p < 0.01.

Pathway enrichment results showed that the upregulated DEGs were mainly involved in ECM‐receptor interactions, the IL‐17 signalling pathway and PI3K‐AKT signalling pathway (Figure 5A), while the downregulated DEGs were involved in the AMPK signalling pathway and cGMP‐PKG signalling pathway (Figure 5B).

The AKT and AMPK signalling pathways play pivotal roles in cell division and cell viability. In order to determine the effect of cold plasma on signalling pathways, phosphorylation of PI3K, AKT and AMPK was investigated by western blot analysis. As shown in Figure 5C, LTPJ increased the phosphorylation of AKT, while the phosphorylation of AMPK was decreased. This suggests that LTPJ plays a role in blocking the AMPK and activating the AKT signal transduction pathway, which may be the reason for the acceleration of wound healing by cold plasma.

### 3.5 LTPJ Accelerated HaCaT Cell Proliferation In Vitro

**FIGURE 6:**
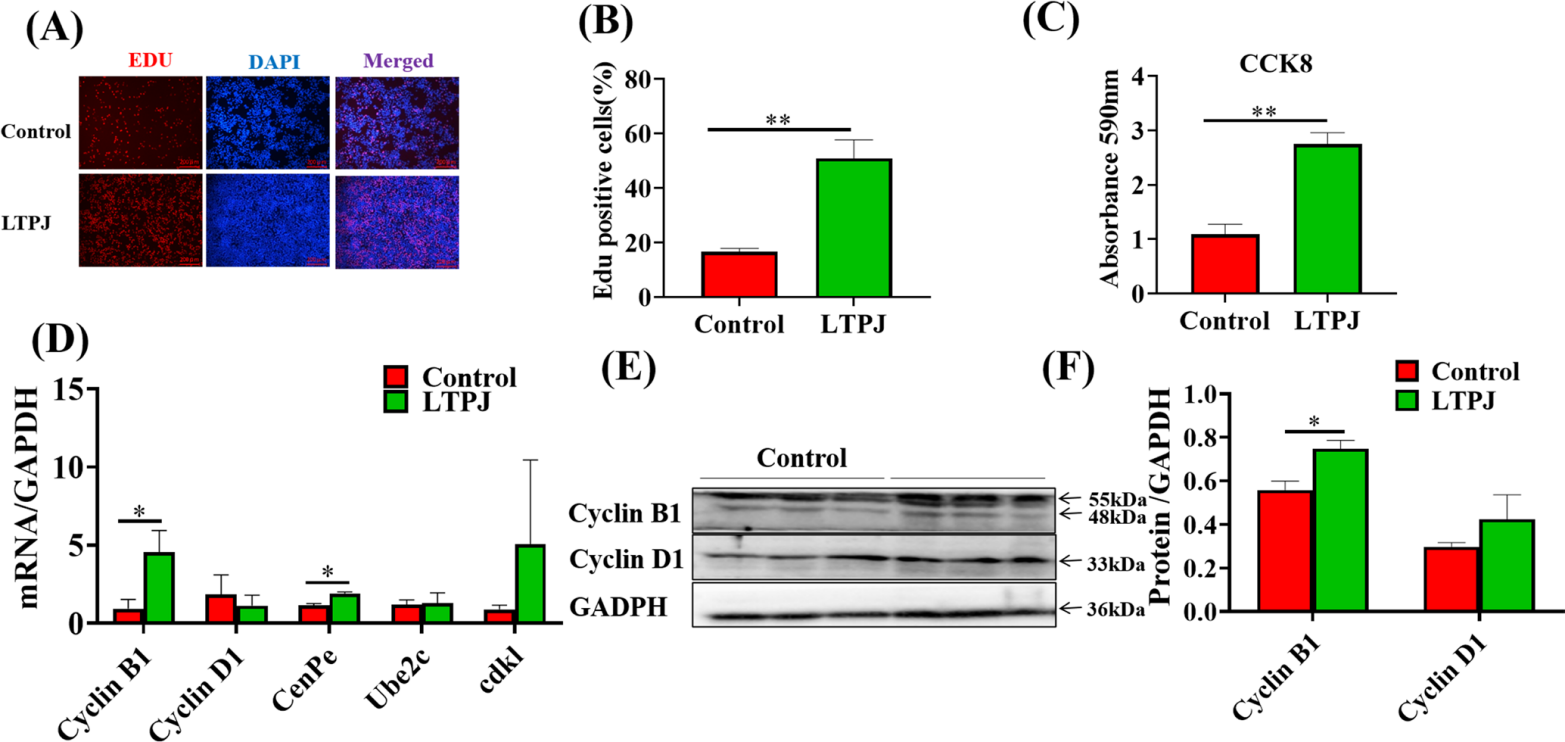
LTPJ promoted the proliferation of HaCaT cells. (A) The proliferation of HaCaT cells was assessed using the EdU assay. Red represents EdU staining, and blue represents cell nuclei counter‐stained with Hoechst 33342. (B) The percentage of EdU‐positive cells was quantified. Quantitative analysis using Image J software. (C) Cell proliferation was quantified using the CCK‐8 assay. (D–F) The expression levels of cyclins B and D were determined. The grouping of blots cropped from different parts of the same gel. The data represent the means ± SD. *n* = 3, *p < 0.05, **p < 0.01.

In addition to the in vivo experiments, we also investigated the impact of LTPJ treatment on the proliferation of HaCaT cells in vitro. HaCaT cells were exposed to LTPJ for 15, 30 and 60 s, which resulted in a significant increase in the proliferation rate of the cells in all three groups (Figure S2A–C). Subsequently, cells were irradiated for 15 s in the later stage of the experiment, and EdU staining revealed that LTPJ increased the proportion of S‐phase cells (Figure 6A,B). The cell counting assay (CCK‐8) demonstrated that LTPJ increased the number of viable cells (Figure 6C). Gene expression analysis showed that LTPJ induced the expression of the cell cycle marker genes cyclin B1 and CenPe (Figure 6D–F).

### 3.6 Effects of LTPJ on PI3K/AKT and AMPK Signalling in HaCaT Cells

**FIGURE 7:**
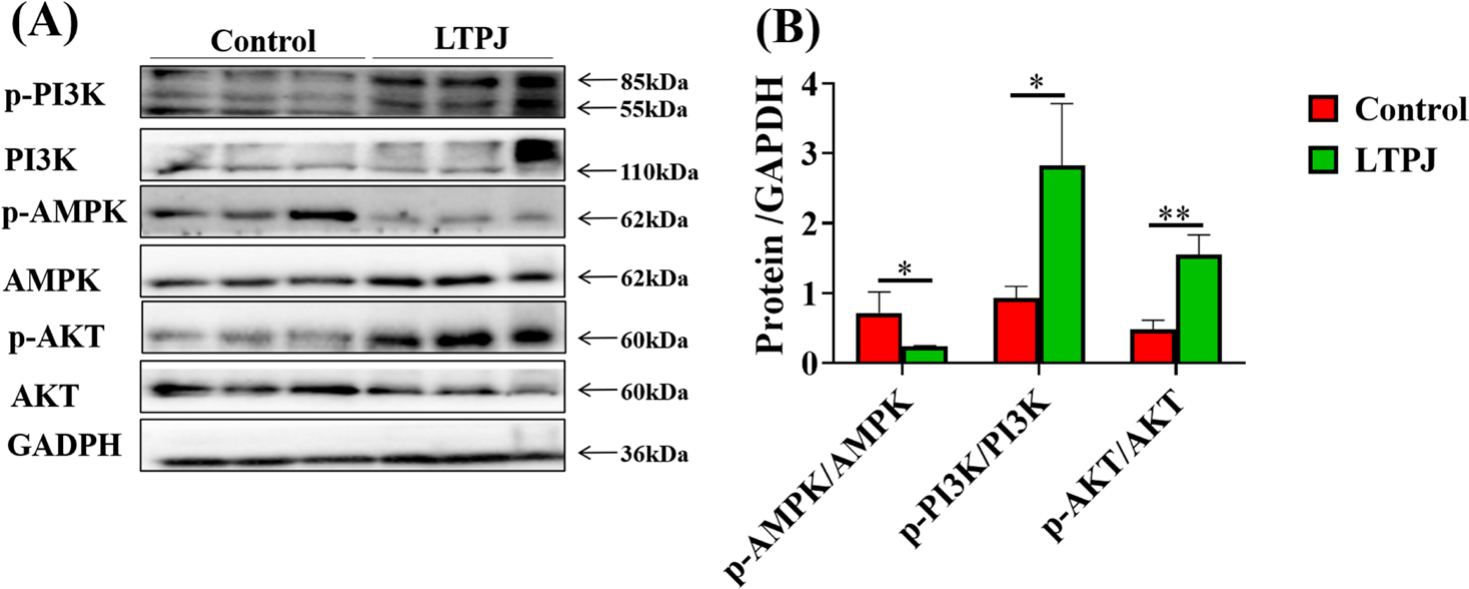
Effect of LTPJ on the activity of relevant signalling pathways. (A) The protein levels of p‐PI3K, PI3K, p‐AKT, AKT, p‐AMPK and AMPK were measured by western blot analysis. (B) Quantification was performed densitometrically using ImageJ software. The grouping of blots cropped from different parts of the same gel. The data represent the means ± SD. *n* = 3, *p < 0.05, **p < 0.01. Low temperature plasma jet (LTPJ) generated by a hand‐held device accelerated skin wound healing in mice model. LTPJ treatment promoted collagen formation in skin wounds. LTPJ treatment increased the activity of PI3K‐AKT pathway and decreased the activity of AMPK pathway both in vivo and in vitro.

To explore the effects of LTPJ treatment on the signalling pathways involved in cell proliferation, we conducted western blot analysis to investigate the phosphorylation of PI3K, AKT and AMPK. As shown in Figure 7A,B, LTPJ increased the phosphorylation of PI3K/AKT and decreased the phosphorylation of AMPK in HaCaT cells.

## 4 Discussion

In this study, a newly designed LTPJ device, which is portable and uses air as the carrier gas, was used to evaluate the effect and mechanism of LTPJ on skin wound healing in mice. Notably, the results demonstrated that LTPJ significantly promoted wound healing and epidermal cell proliferation both in cell culture and in a murine model in vivo. These results were also consistent with previous findings demonstrating that LTPJ has a beneficial effect on the wound healing process. In previous studies, cold plasma promotes skin wound healing by enhancing cell proliferation and migration, as well as antioxidant stress response, or by inhibiting the formation of bacterial biofilms in vitro and in vivo [26, 27]. However, in the present study, LTPJ was found to upregulate the expression cyclin‐dependent protein kinase holoenzyme complex, promoting cell division and viability. Key genes that regulate these biological functions, such as CKS2, CDK1 and CENPE, also exhibited significant increases of transcription levels. CKS2, a cell cycle‐dependent protein kinase subunit, plays a pivotal role in the regulation of somatic cell division [28]. CDK1 and Cyclin B1 are downstream genes of CKS2 and are believed to be crucial regulators in the cell cycle [29, 30]. Numerous researches have reported that Cyclin B1‐CDK1 protein kinase is requisite for the occurrence of mitosis. In another word, cells are incapable of advancing beyond the G2 phase of the cell cycle in the absence of this kinase [31, 32, 33]. The binding of CKS2 to CDK1 through its catalytic subunit has been shown to accelerate the cell cycle [34]. Studies have showed that the depletion of cyclin B1 hinders the initiation of mitosis, further emphasising the crucial role of cyclin B1 as a regulator of mitosis and by extension cell proliferation [33, 35]. During mitosis, centromere protein E (CENPE), a microtubule kinesin, is found at unlinked kinesins and facilitates the movement of individual chromosomes towards the spindle equator through end‐directed microtubule motion [36]. CENPE knockdown at mRNA level results in the suppression of cell proliferation. KIFC1 (HSET) is a Kinesin‐14 family member belonging to type C terminal kinesins [37]. It holds significant importance in the functionality of the mitotic spindle [38]. Studies have revealed that KIFC1 facilitates the proliferation of EC cells by regulating the PI3K/AKT signalling pathway [39]. In this study, both sequencing and real‐time PCR results showed a significant increase in CKS2, CDK1, CENPE and KIFC1, suggesting that LTPJ treatment may promote skin wound healing by activating cell cycles.

In this study, the control group consisted of untreated wounds. If the control group uses pure air treatment, this method may indeed provide a better control in certain situations, as it can reduce the influence of interventions. High airflow may have a drying effect on wounds, but there are no reports indicating its impact on wound healing. However, in many studies, untreated wounds can serve as an effective control group [40, 41], demonstrating that the method used in this paper is also feasible. Furthermore, we will draw on the experiences gained from this experiment to make improvements in our future research.

There are four isoforms of PI3Ks, respectively designated: PI3Kα, PI3Kβ, PI3Kδ and PI3Kγ [42]. They are essential for the activity of PI3K. While PI3Kα is primarily activated by RTKs (receptor tyrosine kinases), PI3Kβ can be activated by either RTKs or downstream GPCRs (G‐protein coupled receptors) through direct interaction with heterotrimeric G protein βγ subunits [43, 44]. AKT, a serine/threonine protein kinase also known as protein kinase B, regulates numerous cellular processes such as cell growth, proliferation and survival. The activation of AKT may initiate by extracellular ligands which turns PI3K pathway on [45]. AKT promotes cell proliferation by activating cell cycle proteins to induce quiescent cells entering the cell cycle [46, 47]. Additionally, studies have demonstrated a positive correlation between cyclin B1 and PI3K/AKT levels [48]. Notably, cyclin B1 was found to regulate this pathway in MDA‐MB‐231 cells treated with AMP [49]. In our study, LTPJ treatment was able to enhance the expression of cyclin B1 and the activity of the PI3K/AKT signalling pathway both in vivo and in vitro, which implied that LTPJ may upregulate cyclin B1 by activating the PI3K/AKT signalling pathway, thereby promoting wound healing and HaCaT cell proliferation.

It is well‐established that AMPK participates in gene transcription, cell mitosis, apoptosis and autophagy. Scientists believed that sustained AMPK activation can hampered cell growth, as well as further promote cell death and apoptosis [49]. The mechanism of AMPK regulating cell growth is by suppression of the mTORC1 pathway [50, 51]. Recent studies found that an energy‐regulated AMPK protein may play a role in activating the proliferation of fibroblasts [49, 52]. As a serine and threonine protein kinase, AMPK plays a pivotal role in regulating energy metabolism. Likewise, AMPK can maintain energy levels by shifting the metabolism of carbohydrates and lipids in the body, as well as respond to the stress stimulation by decreasing ATP consumption and increasing ATP production. It is activated when the ratio of AMP/ATP is low or sustained cellular stress [53]. Metformin, an activator of AMPK, has been reported to suppress cell proliferation in cervical cancer by affecting cell cycles [54]. Our experimental results show that LTPJ could suppress the activity of the AMPK signalling pathway, which may also be one of the reasons for accelerating cell cycles and then promoting the percentage of wound closure.

In conclusion, we have demonstrated that exposure to LTPJ can accelerate the skin wound healing process both in vitro and in vivo. In this study, a comparative transcriptome analysis of skin tissue undergoing wound healing with or without LTPJ treatment was conducted, and multiple differentially expressed genes were identified. In the LTPJ treatment group, the activity of the PI3K/AKT signalling pathway increased while the activity of the AMPK signalling pathway decreased. Our study provides a theoretical basis for future clinical trials of LTPJ wound treatment in humans.

## Conflicts of Interest

The authors declare no conflicts of interest.

## Supporting information


Data S1.

## Data Availability

The data that support the findings of this study are available from the corresponding author upon reasonable request.
