# Supplementary material for: Low Temperature Plasma Jet Treatment Promotes Skin Wound Healing by Enhancing Cell Proliferation via the PI3K‐AKT and AMPK Pathways
Source: Int Wound J. 2025 Feb 11;22(2):e70213. doi: 10.1111/iwj.70213 (PMC11813701; doi:10.1111/iwj.70213)
Supplement: Supplementary file 1 — Data S1. [file IWJ-22-e70213-s001.docx]

| Gene | Primer Sequences |
| --- | --- |
| m-Cenpe | S: 5’AGGATCATGCCACCGAGAAGAC3’ |
|  | A: 5’GCTGTGTCTCTTGGAGTTTCTGG3’ |
| m-Postn | S: 5’CAGCAAACCACTTTCACCGACC3’ |
|  | A: 5’AGAAGGCGTTGGTCCATGCTCA3’ |
| m-Ube2c | S: 5’GGTGACAAAGGAATCTCCGCCT3’ |
|  | A: 5’GGGAGAGTTTGTACCTCAGGTC3’ |
| m-S100a11 | S: 5’GAAGGATGGAAACAACACTCAACT3’ |
|  | A: 5’CGTCACAGTTGAGGTCCAGCTT3’ |
| m-Cks2 | S: 5’ACCCAAAACTCATCTGATGTCCG3’ |
|  | A: 5’GTCGTCTAAAGAGAAGAATATGCG3’ |
| m-Cdk1 | S: 5’CATGGACCTCAAGAAGTACCTGG3’ |
|  | A: 5’CAAGTCTCTGTGAAGAACTCGCC3’ |
| m-Birc5 | S: 5’CCTACCGAGAACGAGCCTGATT3’ |
|  | A: 5’CCATCTGCTTCTTGACAGTGAGG3’ |
| m-Ccl3 | S: 5’ACTGCCTGCTGCTTCTCCTACA3’ |
|  | A: 5’ATGACACCTGGCTGGGAGCAAA3’ |
| m-Col18a1 | S: 5’GGGGAAAGGATTCTTGCCTATG3’ |
|  | A: 5’GAAGGAACAGAGAGTAAACCGTG3’ |
| m-Kifc1 | S: 5’GAGGCCACCTTTGTTGGAAG3’ |
|  | A: 5’ACTTTGGTCACTGCACCCAT3’ |
| m-GAPDH | S: 5’TGCTGAGTATGTCGTGGAGTCT3’ |
|  | A: 5’ATGCATTGCTGACAATCTTGAG3’ |
| m-β-actin | S: 5’GTCCCTGACCCTCCCAAAAG3’ |
|  | A: 5’GCTGCCTCAACACCTCAACCC3’ |
| m-β-tublin | 5’ ATGGTACTGAGCAATGAGCCG3’ |
|  | 5’ GTTCATCATCCACTCAAGGTGT3’ |
| h-Cyclin B1 | S: 5’TCTGGATAATGGTGAATGGACA3’ |
|  | A: 5’CGATGTGGCATACTTGTTCTTG3’ |
| h-Cyclin D1 | S: 5’GGTGAACAAGCTCAAGTGGA3’ |
|  | A: 5’GAGGGCGGATTGGAAATGAA3’ |
| h-Cenpe | S: 5’GGAGAAAGATGACCTACAGAGGC3’ |
|  | A: 5’AGTTCCTCTTCAGTTTCCAGGTG3’ |
| h-Ube2c | S: 5’CTGGCGATAAAGGGATTTCTGCC3’ |
|  | A: 5’GCGAGAGCTTATACCTCAGGTC3’ |
| h-Cdk1 | S: 5’GGAAACCAGGAAGCCTAGCATC3’ |
|  | A: 5’GGATGATTCAGTGCCATTTTGCC3’ |
| h-GAPDH | A: 5’TGCACCACCAACTGCTTAGC3’ |
|  | S: 5’GGCATGGACTGTGGTCATGAG3’ |

TABLE S1 Primers (S, sense; A, antisense) for real time PCR.

m- : murine; h-: human

TABLE S2 Catalogue of primary antibodies for Western blotting

| Name | Length | Host species | Dilution ratio | Brand | Catalogue number |
| --- | --- | --- | --- | --- | --- |
| Anti- Cyclin B1 | 48/55kDa | Rabbit | 1/2000 | abcam | ab181593 |
| Anti- Cyclin D1 | 33kDa | Rabbit | 1/200 | abcam | ab16663 |
| anti-GAPDH(A) | 36KD | Mouse | 1/1000 | arigobio | ARG10112 |
| Phospho- Akt(Ser473) | 60kDa | Rabbit | 1/1000 | CST | #9271 |
| Akt | 60kDa | Rabbit | 1/1000 | CST | #9272 |
| phospho-AMPKα( Thr-172) | 62kDa | Rabbit | 1/2000 | CST | #4188 |
| AMPKα | 62kDa | Rabbit | 1/1000 | CST | #5381 |
| Phospho-PI3 Kinase p85 (Tyr458)/p55 (Tyr199) Antibody | 55/85kDa | Rabbit | 1/1000 | CST | #4228T |
| PI3 Kinase p110α (C73F8) | 110kDa | Rabbit | 1/1000 | CST | #4249T |

TABLE S3 Parameters of low-temperature plasma jet (LTPJ) device

| Control circuit(dead band) | Current | Input Voltage | Duty ratio | Working frequency | Air pump flow rate | Discharge |
| --- | --- | --- | --- | --- | --- | --- |
| 0.88$\mu\text{s}$ | 2.08A | 12.8V | 50% | 120KHz | 5.0L/min | 5000V±5% |

TABLE S4 The weight of mice

| Mouse number | 01 | 02 | 03 | 04 | 05 | 06 | 07 | 08 |
| --- | --- | --- | --- | --- | --- | --- | --- | --- |
| Weight(g) | 21.30 | 22.95 | 21.62 | 21.19 | 21.01 | 21.45 | 21.65 | 22.43 |
| Mean±SD | 21.7±0.62 | | | | | | | |


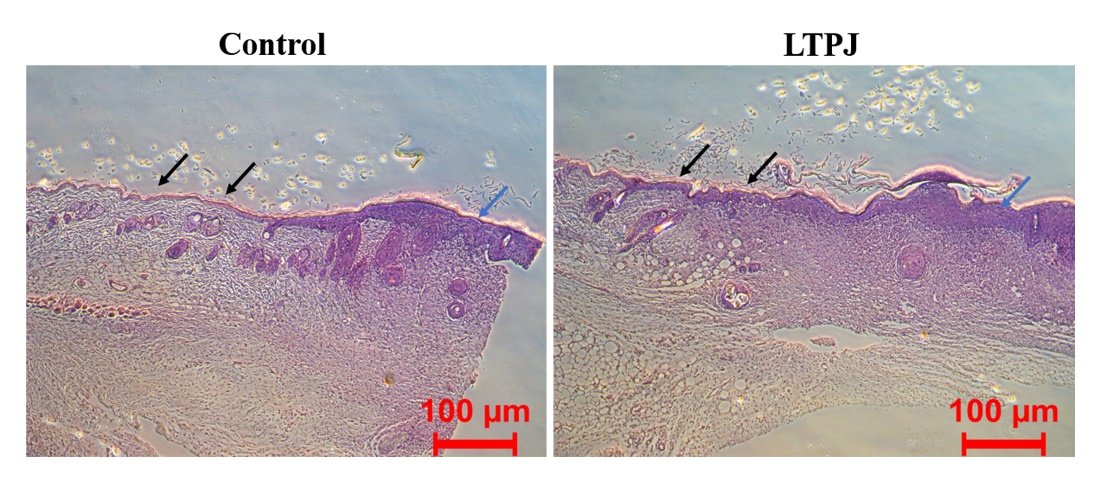


FIGURE S1 HE staining epidermal thickness in control and LTPJ treated wounds on days 8 after the wounding. A larger area of the wound was shown. The black arrow indicates the wound healing site, and the blue arrow indicates the unwounded site.


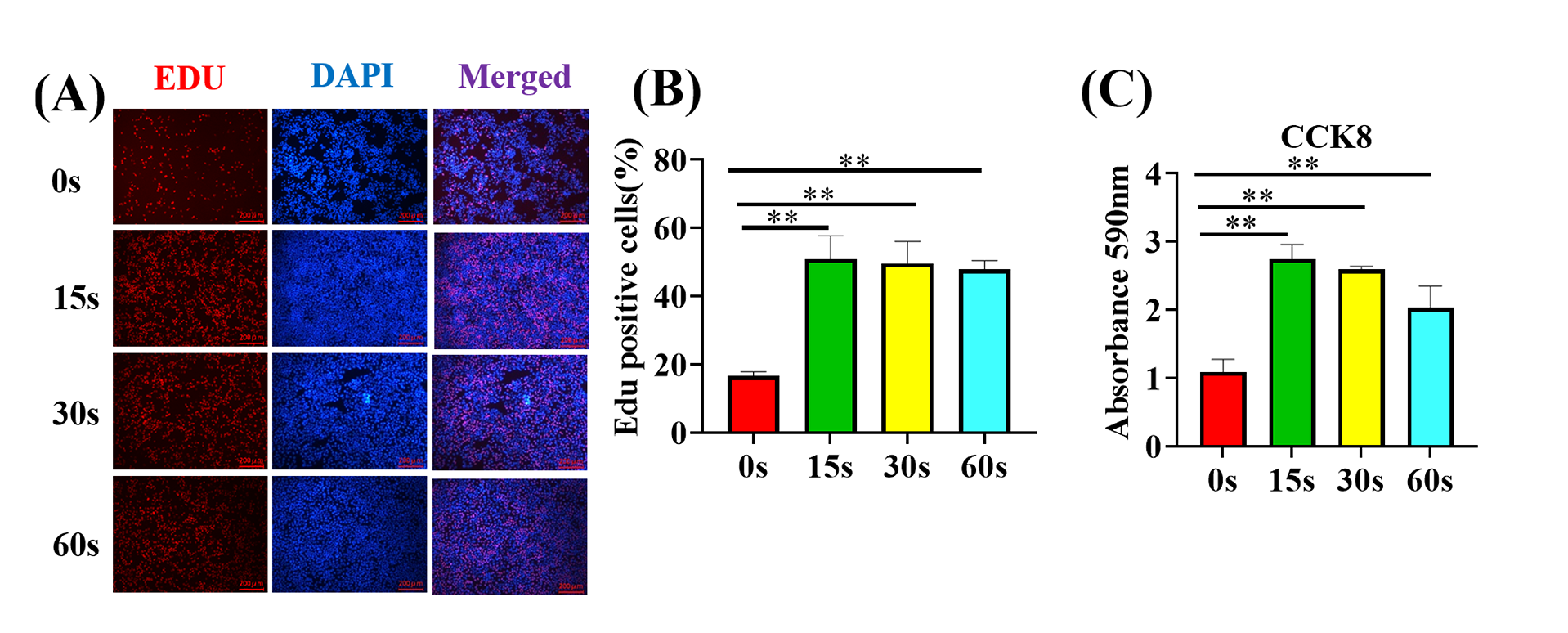


FIGURE S2 The effect of different LTPJ treatment times on the proliferation of HaCaT cells. (A) The proliferation of HaCaT cells was examined using the EdU assay. HaCaT cells were exposed to LTPJ for 15, 30, and 60s. (B) The percentage of EdU-positive cells was quantified. (C) Cell proliferation was quantified using the CCK-8 assay. The data represent the means ± SD. n=3, **p* < 0.05, ***p* < 0.01


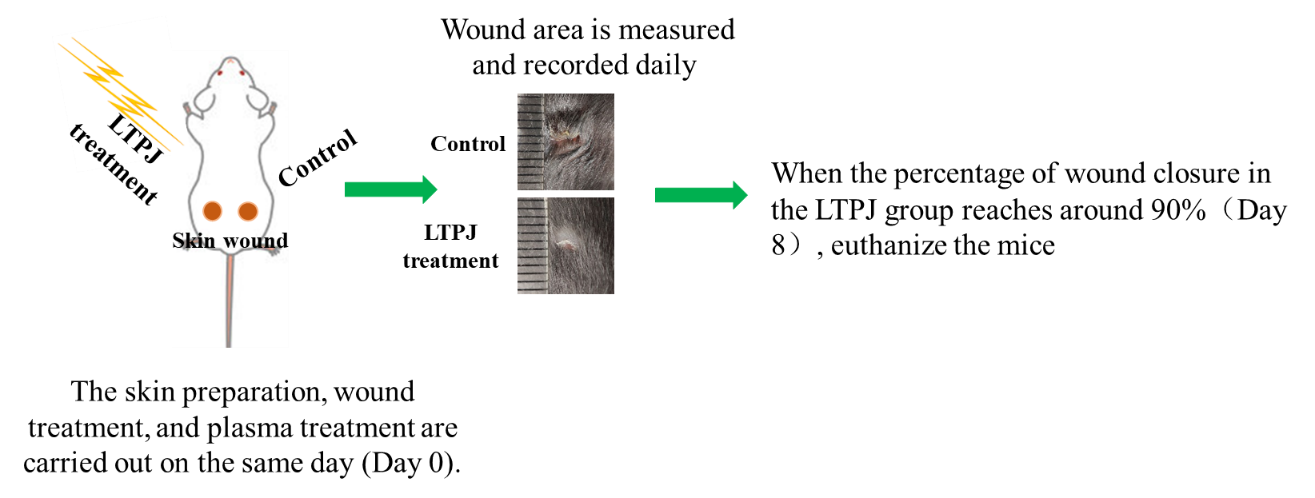


FIGURE S3. The experimental timeline from wounding to sacrifice/tissue harvest.

FIGURE S4. Grayscale value analysis of GAPDH protein expression
